# Failure of total knee arthroplasty with or without patella resurfacing: A study from the Norwegian Arthroplasty Register with 0–15 years of follow-up

**DOI:** 10.3109/17453674.2011.570672

**Published:** 2011-07-08

**Authors:** Stein Håkon Låstad Lygre, Birgitte Espehaug, Leif Ivar Havelin, Stein Emil Vollset, Ove Furnes

**Affiliations:** ^1^The Norwegian Arthroplasty Register, Department of Orthopaedic Surgery, Haukeland University Hospital, Bergen; ^2^Department of Public Health and Primary Health Care, University of Bergen; ^3^Department of Surgical Sciences, University of Bergen, Norway

## Abstract

**Background and purpose:**

Patella resurfacing during primary total knee arthroplasty (TKA) is disputed and new prosthesis designs have been introduced without documentation of their survival. We assessed the impact on prosthesis survival of patella resurfacing and of prosthesis brand, based on data from the Norwegian Arthroplasty Register.

**Patients and methods:**

5 prosthesis brands in common use with and without patella resurfacing from 1994 through 2009 were included n = 11,887. The median follow-up times were 9 years for patella-resurfaced implants and 7 years for implants without patella resurfacing. For comparison of prosthesis brands, also brands in common use with only one of the two treatment options were included in the study population (n = 25,590). Cox regression analyses were performed with different reasons for revision as endpoints with adjustment for potential confounders.

**Results:**

We observed a reduced overall risk of revision for patella resurfaced (PR) TKAs, but the statistical significance was borderline (RR = 0.84, p = 0.05). At 15 years, 92% of PR and 91% of patella non resurfaced (NR) prostheses were still unrevised. However, PR implants had a lower risk of revision due to pain alone (RR = 0.1, p < 0.001), but a higher risk of revision due to loosening of the tibial component (RR = 1.4, p = 0.03) and due to a defective polyethylene insert (RR = 3.2, p < 0.001).

At 10 years, the survival for the reference NR brand AGC Universal was 93%. The NR brands Genesis I, Duracon, and Tricon (RR = 1.4–1.7) performed statistically significantly worse than NR AGC Universal, while the NR prostheses e.motion, Profix, and AGC Anatomic (RR = 0.1–0.7), and the PR prostheses NexGen and AGC Universal (RR = 0.4–0.5) performed statistically significantly better. LCS, NexGen, LCS Complete (all NR), and Tricon, Genesis I, LCS, and Kinemax (all PR) showed no differences in this respect from the reference brand. A lower risk of revision (crude) was found for TKAs performed after 2000 as compared to those performed earlier (RR = 0.8, p = 0.001).

**Interpretation:**

Although revision risk was similar for PR and NR TKAs, we found important differences in reasons for revision. Our results also indicate that survivorship of TKAs has improved.

Use of a patellar component (patella resurfacing) during primary total knee arthroplasty (TKA) is still disputed. The search for improvements has resulted in the introduction of several new designs that are widely used nowadays, although there is no documentation about their survival.

The question of whether or not primary patella resurfacing should be recommended has led to several observational studies, randomized clinical trials (RCT), and meta-analyses (Forster 2004, Parvizi et al. 2005, Nizard et al. 2005, Pakos et al. 2005) and review articles (Meneghini 2008). In a critical appraisal of the available evidence, Calvisi et al. (2009) were not able to find any clear superiority between either of the two treatments due to methodological limitations in the published studies. Studies based on data from arthroplasty registers have found a higher risk of revision when the patella was left untreated (Furnes et al. 2002, the Swedish Knee Arthroplasty Register Annual Report 2009, Clements et al. 2010). Furnes et al. 2002) found that the increased revision risk was mainly related to revisions due to pain. Some recent studies have, however, indicated that there is no difference in patients' perception of postoperative pain in the two groups of treatment (Johnston et al. 2009, Lygre et al. 2010) and that the observed differences in risk of revision due to pain may be caused by the exclusive option of a secondary patella resurfacing of the originally patella unresurfaced knee. This is supported by a recent study from the Australian Orthopaedic Association National Joint Replacement Registry (AOANJRR) that suggested that surgeons may be more inclined to revise a patella non resurfaced implant knee with a secondary patella resurfacing if the patient presents later with knee pain, given that the option is still available (Clements et al. 2010)

Few studies have compared survival of different prosthesis brands and implant designs, but a previous study (Furnes et al. 2002) from the Norwegian Arthroplasty Register (NAR) did not find any statistically significant short-term differences in revision rates between the most commonly used brands in Norway. Other national arthroplasty registers with longer follow-up have reported statistically significant differences between some commonly used brands in their annual reports (Australian Orthopaedic Association National Joint Replacement Registry Annual Report 2009, The Swedish Knee Arthroplasty Register Annual Report 2009).

Based on data in the NAR, we compared overall survival of cemented knee prostheses with and without resurfacing of the patella, and assessed the survival of some widely used TKA brands.

## Materials and methods

### The Norwegian Arthroplasty Register (NAR)

The NAR was started as a register for total hip arthroplasty in 1987 (Havelin 1999, Havelin et al. 2000) and had been extended to include all artificial joint replacements by January 1994 (Havelin et al. 2000). Information on primary knee arthroplasties and revisions is reported on a standardized form by the orthopedic surgeon and individual reports on TKAs performed have since been received from 82 orthopedics departments. Practically all TKAs (99%) are reported to the NAR (Espehaug et al. 2006). The unique identification number assigned to each resident of Norway is used to link information on revisions. A revision is defined as surgical removal or exchange of a part of or the whole implant, or as insertion of a patellar component.

### Study sample

**Figure 1.**
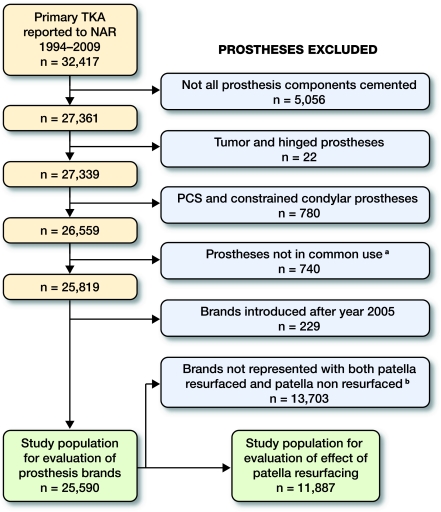
Description of the selection procedure.

By December 10, 2009, 32,417 primary TKAs had been reported to the NAR. Only TKAs with all components fixed with cement were included in the present study. This was because the use of cement was most common (n = 27,361, 84%) and it was to make the results more comparable to results from other studies. We excluded hinged prostheses (n = 22) and prostheses with posterior cruciate ligament sacrificing design (except for the LCS mobile bearing) or constrained condylar design (n = 780). Furthermore, patella resurfaced (PR) implants of a specific brand were excluded if used in less than 200 operations (n = 354). This also applied to brand-specific patella non resurfaced (NR) implants (n = 386). In addition, we did not include prosthesis brands introduced later than 2005 (n = 229), leaving 25,590 prostheses in the study population for comparison of the survival of prosthesis brands (Figure 1). Where possible, prosthesis brands were categorized as PR and NR (Table 1).

**^a^** Patella resurfaced implants of a specific brand were excluded if used in less than 200 operations (n=354). This also applied to brand specific patella non resurfaced implants (n=386).
**^b^** For comparison of patella resurfaced and patella non resurfaced TKAs the material was further restricted to those brands with both patella resurfaced and patella non resurfaced TKAs represented with larger numbers than 200 (Tricon, Genesis I, AGC, LCS and NexGen).

**Table 1.** Characteristics of primary total knee arthroplasties reported to the Norwegian Arthroplasty Register from 1994 to 2009

		Numbers of hospitals			Men	< 60 years	Median	OA**^a^**	PO **^b^**	MB **^c^**
Prosthesis	n	n	n > 50	n > 100	%	%	age	%	%	%
Patella resurfacing **^d^**	11,887	70	46	37	28	13	72	81	26	34
Patella resurfaced	2,547	51	12	6	28	16	72	71	27	21
Patella non resurfaced	9,340	69	41	32	28	13	73	83	25	38
Prosthesis brand	25,590	79	61	53	31	15	72	84	27	34
NR AGC Universal	2,123	31	11	7	31	12	73	86	22	0
NR Tricon **^e^**	633	23	5	1	22	7	73	75	24	0
NR Genesis I	2,304	28	13	7	26	12	73	75	26	0
NR LCS **^f, g^**	3,526	36	15	10	28	13	72	88	26	100
NR Duracon **^h^**	1,283	18	10	5	33	14	71	87	30	0
NR NexGen	754	18	4	2	35	17	71	87	26	0
NR Profix	6,304	40	25	20	31	16	71	85	25	0.2
NR LCS Complete	4,090	36	15	13	33	16	70	90	30	100
NR e.motion	434	4	2	1	33	18	70	94	40	100
NR AGC Anatomic	1,298	18	4	4	37	15	70	88	26	0
PR AGC Universal	425	21	3	1	24	13	73	66	25	0
PR Tricon **^e^**	392	21	3	0	26	21	71	54	29	0
PR Genesis I	704	23	5	1	25	14	71	72	28	0
PR LCS **^i^**	532	12	2	1	27	13	73	92	23	100
PR Kinemax	294	12	2	0	19	9	74	87	22	0
PR NexGen	494	11	1	1	36	21	69	64	28	0

**^a^** Primary osteoarthritis of the knee (%).

**^b^** Previously operated in the knee (%).

**^c^** Mobile-bearing prostheses (%).

**^d^** Study population restricted to brands represented by both patella resurfaced and patella non resurfaced prostheses.

**^e^** Tricon C or Tricon M femoral component used on the femoral side and Tricon II used on the tibial side.

**^f^** 375 of the primary patella non resurfaced LCS prostheses were used in combination with an LCS Universal tibial component.

**^g^** 3032 with mobile-bearing rotating platform system and 494 with mobile-bearing meniscal system.

**^h^** One Duracon prosthesis had an all-polyethylene tibial component.

**^i^** 323 with mobile-bearing rotating platform system and 209 with mobile-bearing meniscal system.

For comparison of PR and NR TKAs, the material was further restricted to those brands with both PR and NR TKAs that were represented by larger numbers than 200 (n = 11,887) (Tricon, Genesis I, AGC, LCS, and NexGen).

### Statistics

We used survival analysis with revision of the implant as endpoint. Information on deaths and emigrations was retrieved from Statistics Norway, Oslo, until December 10, 2009. The survival times of implants in patients who had died or emigrated without revision of the prosthesis were censored at the date of death or emigration. Otherwise, the survival times were censored at the end of the study on December 10, 2009.

The reversed Kaplan-Meier method was used to calculate the median follow-up (Schemper and Smith 1996). To evaluate the effects of patella resurfacing and of prosthesis brands, relative risk (RR) estimates from Cox regression models were obtained. Adjustments were performed for possible confounding by age group (< 60 years, 60–70 years, > 70 years), sex, previous operation of the knee (operated or not), diagnosis (primary osteoarthritis of the knee, other) and prosthesis brand. The covariate age group was represented with indicator variables since the assumption of a log-linear relationship between age and the revision rate was not justified. The impact of patella resurfacing was further assessed with different causes of revision as endpoint. More than one reason for revision could be reported by the surgeon. Pain was, however, only considered as a reason for revision when not reported in combination with other causes. In some of these analyses, adjustment for prosthesis brand was done by stratification when one or more of the brands was without failures (periprosthetic fracture, dislocation of the patella, dislocation (not the patella), and defective polyethylene insert). Analogous survival curves for these specific endpoints are presented without adjustments for brands.

Patella non resurfaced TKAs were used as the reference when assessing the impact of patella resurfacing, while the NR AGC Universal was used as reference when comparing prosthesis brands. The latter was chosen because NR AGC Universal had been used throughout the study period in large numbers. The AGC prosthesis has also been used as reference in reports from the Swedish Knee Arthroplasty Register (the Swedish Knee Arthroplasty Register Annual Report 2009) and its results have been reported with favorable survival rates with long-term follow-up (97.8% overall survival at 20 years) (Ritter 2009). Tests and inspections of plotted Schoenfeld residuals (Grambsch et al. 1995) were performed to investigate whether the proportional hazards assumption of the Cox models was valid. The assumption was found to be valid for comparison of PR and patella NR prostheses when using any revision as endpoint (p = 1.0) and when using specific reasons as endpoint (p = 0.07–0.9). Regarding comparison of prosthesis brands, the assumption was valid for all brands except for PR Tricon, PR Kinemax, and NR Tricon. Survival curves for the adjusted percentage of unrevised implants were estimated with treatment option as stratification factor. Survival percentages at 5, 10, and 15 years are presented in the tables. Curves and percentages are given for survival times when more than 50 implants remained at risk of revision.

**Figure 2.**
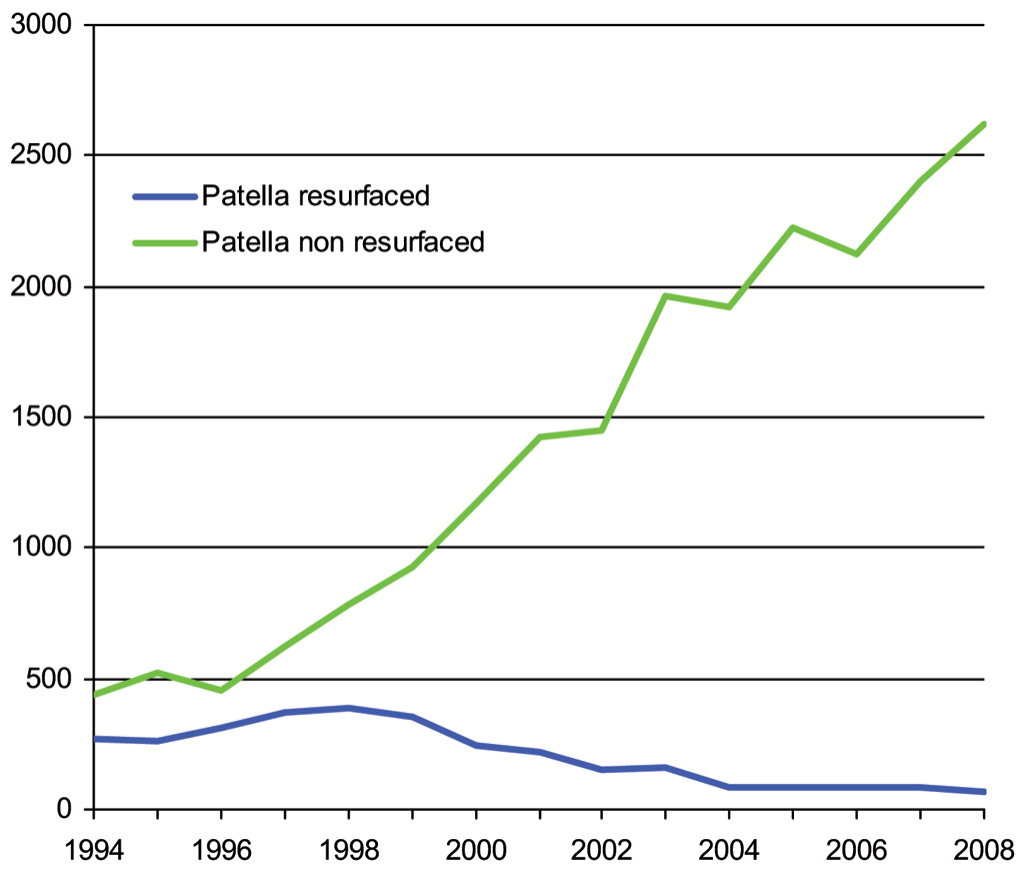
Time trends in the use of cemented patella resurfaced and patella non resurfaced TKAs in Norway, 1994–2008. 2009 was not included since follow-up was only until December 10, 2009.

Since use of patella resurfacing (Figure 2) and prosthesis brands (Table 2) changed throughout the study period, survival was also compared within 2 separate time periods, namely for operations performed from 1994 through 2000 (with follow-up until December 10, 2009) and from 2001 to December 10, 2009. Comparisons within each time period were only performed when the number of available prostheses exceeded 200.

**Table 2.** Follow-up and numbers at risk by years after operation

Prosthesis	1994–2009					1994–2000					2001–2009		
	Median follow-up (years)	Number at risk by years after operation				Median follow-up (years)	Number at risk by years after operation				Median follow-up (years)	Number at risk by years after op.	
		0	5	10	15		0	5	10	15		0	5
Patella resurfacing **^a^**													
Patella resurfaced	9.1	2,547	1,930	986	76	11	1,831	1,519	986	76	6.0	716	411
Patella non resurfaced	7.2	9,340	6,522	1957	116	10	4,255	3,506	1,957	116	5.8	5,085	3,016
Prosthesis brand													
NR AGC Universal	6.9	2,123	1,445	376	14	9.8	841	708	376	14	5.7	1,282	737
NR Tricon	12	633	494	341	66	12	633	494	341	66		–	–
NR Genesis I	9.6	2,304	1,854	913	35	10	1,946	1,581	913	35	7.9	358	273
NR LCS	6.6	3,526	2,573	323	1	9.7	827	716	323	1	6.0	2,699	1,857
NR Duracon	1.7	1,283	248	118	4	11	204	173	118	4	1.5	1,079	75
NR NexGen	3.2	754	156	4	–	10	8	7	4	–	3.2	746	149
NR Profix	4.4	6,304	2,576	51	–	9.2	320	271	51	–	4.2	5,984	2,305
NR LCS Complete	1.9	4,090	59	–	–	–	–	–	–	–	1.9	4,090	59
NR e.motion	4.1	434	76	–	–	–	–	–	–	–	4.1	434	76
NR AGC Anatomic	2.7	1,298	152	15	–	10	31	25	15	–	2.7	1,267	127
PR AGC Universal	9.0	425	353	160	4	10	309	257	160	4	7.2	116	96
PR Tricon	13	392	313	226	67	13	392	313	226	67	–	–	–
PR Genesis I	11	704	581	396	5	11	672	557	396	5	7.2	32	24
PR LCS	8.9	532	446	150	–	9.9	346	293	150	–	7.5	186	153
PR Kinemax	10	294	240	138	30	11	265	214	138	30	7.2	29	26
PR NexGen	4.9	494	237	54	–	10	112	99	54	–	4.0	382	138

**^a^** Study population restricted to brands represented with both patella resurfaced and patella non resurfaced prostheses.

We considered p-values of less than 0.05 to be statistically significant. The statistical software programs R version 2.10.1 (The R Foundation for Statistical Computing) and SPSS version 17.0 were used.

## Results

Of the 11,887 prostheses included when evaluating resurfacing of the patella, 786 (6.6%) were revised by the end of the study period while this applied to 1,204 (4.7%) of the 25,590 prostheses included when evaluating brands. The distributions of age, sex, and patients previously operated in the knee were similar for patients with a PR TKA and those who had a patella NR TKA. Patella NR TKAs were performed in more hospitals and were more likely to have had primary osteoarthritis of the knee as diagnosis (Table 1). Among brands, differences were observed for most of the baseline characteristics of the patients (Table 1). Median follow-up and number at risk 0, 5, 10, and 15 years after the operation are given in Table 2.

### Patella resurfacing (Table 3 and Figure 3)

**Table 3a.** Cox relative revision risk (RR) and survival percentages, estimated with all causes of revision as endpoint

Prosthesis	1994–2009								
	n	Revised	RR	95% CI	p	5 yr	10 yr	15 yr	95% CI**^a^**
Patella resurfacing**^b,c^**									
Patella non resurfaced	9,340	614	1			94.8	93.0	91.4	90.4–92.4
Patella resurfaced	2,547	172	0.84	0.71–1.00	0.052	95.9	94.3	92.1	90.7–93.6
Prosthesis brands **^d^**									
NR AGC Universal	2,123	120	1			94.9	93.2	–	91.9–94.5
NR Tricon	633	72	1.67	1.24–2.24	0.001	93.4	88.6	85.0	81.7–88.3
NR Genesis I	2,304	222	1.43	1.14–1.79	0.002	92.4	90.4	–	89.1–91.6
NR LCS	3,526	177	0.84	0.67–1.06	0.14	95.8	94.1	–	93.2–95.1
NR Duracon	1,283	56	1.45	1.05–1.99	0.02	94.5	90.6	–	87.4–93.8
NR NexGen	754	23	0.79	0.51–1.24	0.3	95.7	–	–	93.9–97.5
NR Profix	6,304	195	0.66	0.52–0.82	< 0.001	96.7	95.7	–	94.9–96.5
NR LCS Complete	4,090	111	0.94	0.72–1.22	0.6	95.4	–	–	94.2–96.6
NR e.motion	434	2	0.09	0.02–0.37	0.001	99.5	–	–	98.9–100
NR AGC Anatomic	1,298	31	0.66	0.45–0.99	0.04	97.1	–	–	95.9–98.2
PR AGC Universal	425	14	0.48	0.27–0.83	0.009	97.2	96.6	–	94.9–98.4
PR Tricon	392	44	1.28	0.90–1.82	0.2	95.9	92.7	87.5	83.9–91.2
PR Genesis I	704	62	1.17	0.86–1.59	0.3	94.0	92.0	–	90.0–94.0
PR LCS	532	41	1.23	0.86–1.75	0.3	93.1	91.7	–	89.1–94.2
PR Kinemax	294	23	1.14	0.73–1.78	0.6	95.6	92.6	–	89.3–95.8
PR NexGen	494	11	0.40	0.22–0.74	0.004	98.6	96.7	–	94.6–98.8

**^a^** Confidence interval for last reported survival percentage.

**^b^** Cox relative risk estimates and survival percentages with adjustment for age group, sex, diagnosis, previous operation of the knee, and prosthesis brand.

**^c^** Study population restricted to brands represented by both patella resurfaced and patella non resurfaced prostheses.

**^d^** Cox relative risk estimates and survival percentages with adjustment for age group, sex, diagnosis, and previous operation of the knee.

**Table 3b.** Cox relative revision risk (RR) and survival percentages, estimated with all causes of revision as endpoint

Prosthesis	1994–2000 **^a^**				2001–2009			
	n	Revised	RR	p	n	Revised	RR	p
Patella resurfacing **^b,c^**								
Patella non resurfaced	4,255	349	1		5,085	265	1	
Patella resurfaced	1,831	138	0.84	0.09	716	34	0.99	1.0
Prosthesis brands **^d^**								
NR AGC Universal	841	51	1	–	1,282	69	1	–
NR Tricon	633	72	1.84	0.001	0	–	–	–
NR Genesis I	1,946	175	1.46	0.02	358	47	2.01	< 0.001
NR LCS	827	50	0.90	0.6	2,699	127	0.81	0.1
NR Duracon	204	18	1.41	0.2	1,079	38	1.39	0.1
NR NexGen	8	1	–	–	746	22	0.72	0.2
NR Profix	320	12	0.59	0.1	5,984	183	0.62	0.001
NR LCS Complete	0	–	–	–	4,090	111	0.85	0.3
NR e.motion	0	–	–	–	434	2	0.09	0.001
NR AGC Anatomic	31	1	–	–	1,267	30	0.63	0.03
PR AGC Universal	309	13	0.69	0.2	116	1	–	–
PR Tricon	392	44	1.46	0.07	0	–	–	–
PR Genesis I	672	57	1.25	0.2	32	5	–	–
PR LCS	346	20	0.97	0.9	186	21	–	–
PR Kinemax	265	22	1.32	0.3	29	1	–	–
PR NexGen	112	4	–	–	382	7	0.33	0.005

**^a^** Follow-up until December 10, 2009

**^b^** ^–^ **^d^** See Table 3a.

**Figure 3.**
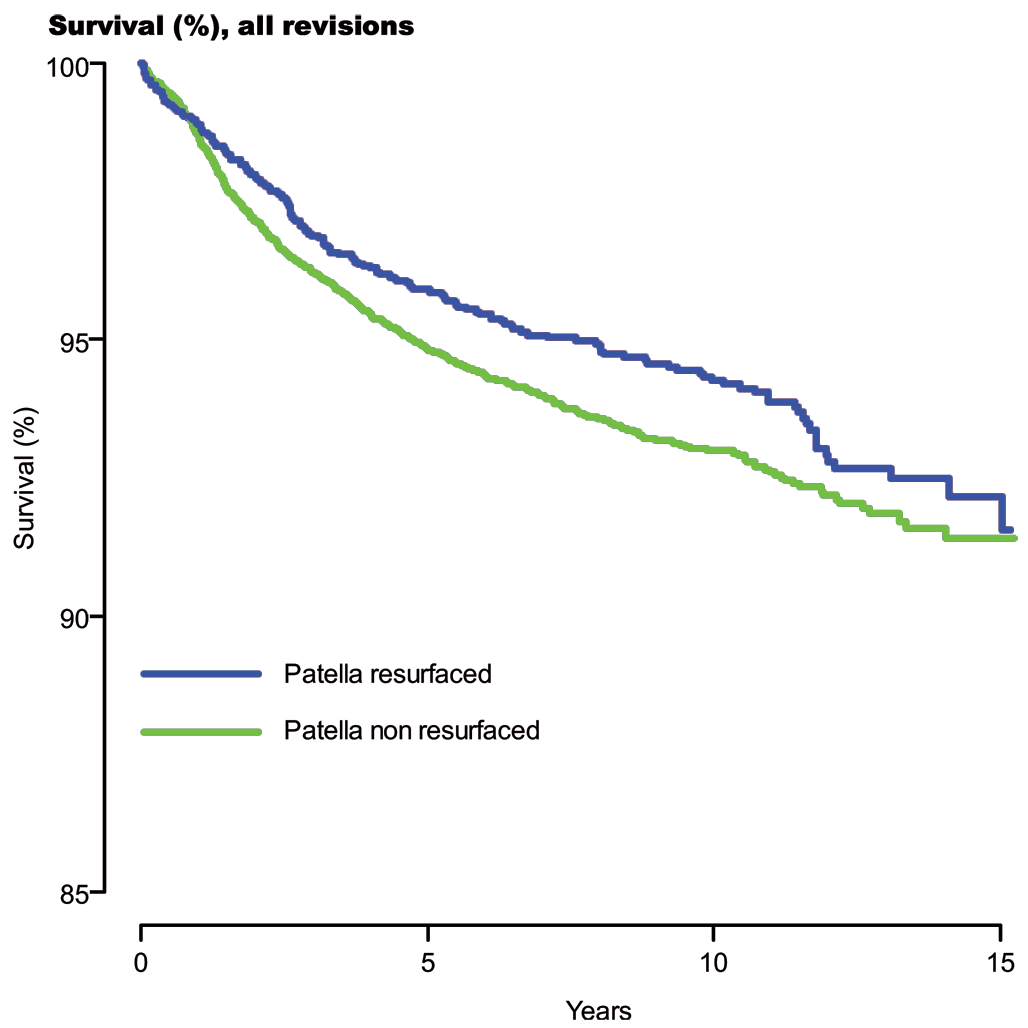
Survival rates (%) for cemented patella resurfaced and patella non-resurfaced TKAs in Norway, 1994–2009; all revisions. Cox regression results with adjustment for age group, sex, diagnosis, previous operation of the knee, and brand of prosthesis.

After 15 years, the overall survival was 92% (CI: 91–94) for PR TKAs and 91% (CI: 90–92) for patella NR TKAs. We found a lower risk of revision for PR TKAs than for NR TKAs (RR = 0.84, CI: 0.71–1.00; p = 0.05). We did not find any statistically significant differences in survival between PR and NR TKAs within each time period.

### Reasons for revision (Table 4 and Figure 4)

**Table 4.** Cox relative revision risk (RR), patella non resurfaced TKAs vs. patella resurfaced primary TKAs by reason for revision, 1994–2009

Reason for revision **^a^**	Revised patella resurfaced	Revised patella non-resurfaced	RR **^b^**	95% CI	p
Loose femur	21	65	0.82	0.49–1.39	0.5
Loose tibia	57	149	1.42	1.03–1.95	0.03
Loose patella	13				
Dislocation, patella	7	22	0.98	0.42–2.32	1.0
Dislocation, other	4	12	2.22	0.71–6.95	0.2
Instability	30	73	0.96	0.61–1.50	0.9
Malalignment	17	43	1.18	0.65–2.12	0.6
Deep infection	41	93	1.32	0.90–1.95	0.2
Periprosthetic fracture	7	20	1.53	0.64–3.70	0.3
Defect polyethylene ins.	30	16	3.23	1.71–6.11	< 0.001
Pain alone	8	202	0.12	0.06–0.23	<0.001

**^a^** More than one reason for revision may have been reported.

**^b^** Cox relative risk of revision estimates (RR) adjusted for age group, sex, diagnosis, previous operation of the knee, and brand (AGC Universal, Tricon, Genesis I, LCS, and NexGen) are reported for patella resurfaced vs. patella non resurfaced prostheses.

**Figure 4.**
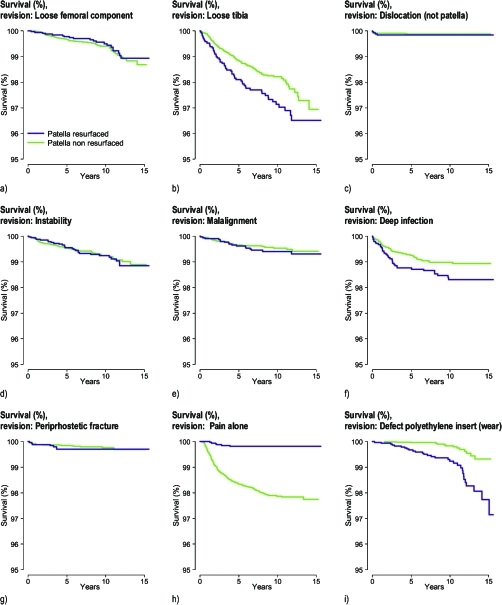
Survival rates (%) for cemented patella resurfaced and patella non resurfaced TKAs in Norway, 1994–2009; specific reasons for revision. Cox regression results with adjustment for age group, sex, diagnosis, previous operation of the knee, and prosthesis brand [(c), (g), (i) without adjustment for brand].

For most of the reasons for revision registered, we did not find any statistically significant differences in survival between PR and NR TKAs. However, PR TKAs had a statistically significantly lower risk of revision due to pain alone (RR = 0.1, CI: 0.1–0.2; p < 0.001) (Table 4 and Figure 4h), but there was a statistically significantly higher risk of revision due to loosening of the tibial component (RR = 1.4, CI: 1.0–2.0; p = 0.03) and due to a defective polyethylene insert or wear (RR = 3.2, CI: 1.7–6.1; p < 0.001).

The lower risk of revision due to pain alone for PR prostheses was also found when restricting the material to TKAs performed from 1994 through 2000 (RR = 0.04, CI: 0.01–0.14; p < 0.001) but not to those performed from 2001 through December 10, 2009 (RR = 0.7, CI: 0.3–1.8; p = 0.4). Higher risk of revision due to loosening of the tibial component for PR prostheses was also found to be statistically significant for TKAs performed in the first time period (RR = 1.5, CI: 1.0–2.2; p = 0.04) but not in the last (RR = 1.6, CI: 0.9–2.9; p = 0.14). Higher risk of a defective polyethylene insert for PR prostheses was also found for TKAs performed in the first time period (RR = 3.6, CI: 1.9–6.8; p < 0.001) while a total of only two events of failure was observed in the last time period. There was also a tendency for higher risk of revision for PR TKAs due to deep infection in the first time period (RR = 1.6, CI: 1.0–2.5; p = 0.06) but not in the last time period (RR = 0.7, CI: 0.3–1.8, p = 0.45).

### Prosthesis brands (Table 3, Figure 5, and Figure 6)

**Figure 5.**
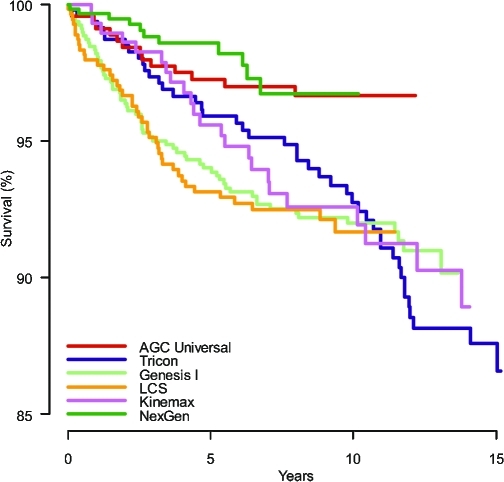
Survival rates (%) for cemented patella resurfaced prosthesis brands in Norway, 1994–2009; all revisions. Cox regression results with adjustment for age group, sex, diagnosis, and previous operation of the knee.

**Figure 6.**
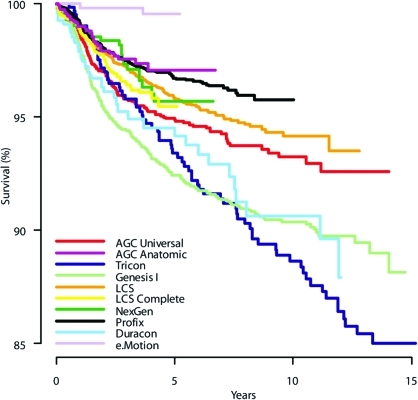
Survival rates (%) for cemented patella non resurfaced prosthesis brands in Norway, 1994–2009; all revisions. Cox regression results with adjustment for age group, sex, diagnosis, and previous operation of the knee.

At 10 years, the survival for the reference brand—patella NR AGC Universal—was 93% (CI: 92–95). We observed some variability in survival among prosthesis brands at 10 years of follow-up, ranging from 89% for NR Tricon to 97% for PR NexGen (RR = 0.2, CI: 0.1–0.45; p ≤ 0.001). For brands represented by both PR and NR TKAs (Tricon, Genesis I, AGC Universal, LCS, and NexGen), the PR TKAs had higher percentage survival than the same brand of NR TKAs, except for the LCS where NR TKAs showed higher survival.

Compared to the reference brand, NR Tricon (RR = 1.7, CI: 1.2–2.2; p = 0.001), NR Genesis I (RR = 1.4, CI: 1.1–1.8; p = 0.002) and NR Duracon (RR = 1.4, CI: 1.1–2.0; p = 0.02) performed statistically significantly worse while NR Profix (RR = 0.7, CI: 0.5–0.8; p < 0.001), NR e.motion (RR = 0.1, CI: 0.0–0.4; p = 0.001), NR AGC Anatomic (RR = 0.7, CI: 0.4–1.0; p = 0.04), PR AGC Universal (RR = 0.5, CI: 0.3–0.8; p = 0.009) and PR NexGen (RR = 0.4, CI: 0.2–0.7; p = 0.004) performed significantly better.

A separate analysis performed on patella resurfaced LCS prostheses did not show any advantage of the mobile bearing rotating platform system (n = 323) compared to the mobile bearing meniscal system (n = 208) (RR = 1.3, 95% CI: 0.6–2.4; p = 0.5). A corresponding analysis performed on patella NR LCS prostheses did not show any advantage of the mobile bearing rotating platform system (n = 3,031, 375 with LCS Universal tibial insert) compared to the mobile bearing meniscal system (n = 477) (RR = 0.9, CI: 0.6–1.4; p = 0.7).

A significantly lower risk of revision was found when comparing crude survival of the TKAs performed in the last time period to those performed in the first (RR = 0.8, CI: 0.7–0.9; p = 0.001).

### Patient-related factors

We found no statistically significant difference in survival of TKAs in women as compared to men (RR = 1.0, CI: 0.9–1.1; p = 1.0) and for knees with osteoarthritis as compared to other diagnoses (RR = 0.9, CI: 0.8–1.1; p = 0.3). Knee prostheses in older patients (> 70 years) were found to perform better than in younger patients (< 60 years) (RR = 0.4, CI: 0.3–0.4; p < 0.001), and previously operated knees performed more poorly than those that were not previously operated (RR = 1.3, CI: 1.1–1.4; p < 0.001).

## Discussion

### Summary

We observed a statistically non-significant reduced risk of revision of 16% for PR TKAs as compared to patella NR TKAs based on data with 0–15 years of follow-up in the NAR. There were, however, statistically significant differences in reasons for revision. While patella NR TKAs were more often revised due to pain, PR TKAs had a higher risk of revision due to aseptic loosening of the tibial component and due to a defective polyethylene insert or wear when assessed with long follow-up.

Regarding brands, NR Tricon, NR Genesis I, and NR Duracon performed significantly worse than the reference brand NR AGC Universal, with about 1.5 times higher risk of revision. NR Profix, NR e.motion, NR AGC Anatomic, PR AGC Universal, and PR NexGen had a significantly reduced risk of revision. NR LCS, NR NexGen, and NR LCS Complete performed similarly to the reference brand, as did PR Tricon, PR Genesis I, PR LCS, and PR Kinemax. For brands that were evaluated both with and without patella resurfacing, patella resurfacing appeared to offer the best survival performance, except for the LCS design were patella resurfacing had the poorest survival.

### Explanations/mechanisms

The (non–significantly) higher risk of revision for patella NR TKAs was found to be caused mainly by more revisions due to pain only. A recent study from the NAR (Lygre et al. 2010) and a high-powered multicenter RCT including 1,715 patients (Johnston et al. 2009) could not, however, demonstrate any difference in level of pain between the two treatment groups. One explanation could be the exclusive option of secondary resurfacing of the patella for patella NR TKAs. Hence, lower risk of revision due to pain may not necessarily be caused by less severe pain but because surgeons may be more likely to revise a painful patella NR TKA than a painful PR TKA.This explanation is also supported in a recent study from the AOANJRR (Clements et al. 2010). Our findings also indicate that the difference in risk of revision due to pain was greatest for TKAs performed in the first time period (1994 to 2001). This might be due to the introduction of the newer and more patella-friendly implant NexGen and due to the stop in use of some older inferior designs that were represented in our material by Genesis I and Tricon. The NexGen implant has also been found to perform statistically significantly better regarding pain than the implants AGC Universal and Genesis I in a recent study from the NAR (Lygre et al. 2010).

The rate of aseptic loosening of the tibial component for PR TKAs has been found to be highest with long follow-up, and may also be associated with wear of the extra polyethylene element on the patellar side (Ogon et al. 2002). It might thus be explained by a higher volume of polyethylene particles in the joint (Goodman and Lidgren 1992). Such an association is also supported by similar phenomena observed when assessing wear and loosening of cups after hip arthroplasty (Wroblewski et al. 2004, Wilkinson et al. 2005). Patella resurfacing might also increase the patellofemoral offset due to conservative bone resection. The resulting increased forces over the patellofemoral joint and onto the tibia might thereby increase tibial loosening and wear of the tibial polyethylene insert. This is also supported by the observation of higher risk of revision due to defective polyethylene inserts in PR TKAs apparent after about 10 years of follow-up.

Regarding brand-specific results, the good performance of the NR e.motion prosthesis and also the inferior results of the PR LCS prosthesis as compared to the NR LCS, are interesting but could be caused by variables other than the implant itself—since the use of NR e.motion and PR LCS was restricted to few hospitals. However, the revision rate for the PR LCS prosthesis has also been reported to be higher then anticipated in operations reported to the AOANJRR (Australian Orthopaedic Association National Joint Replacement Registry Annual Report 2009). Higher failure rates were found to be associated with the use of a metal-backed LCS patellar component as compared to an all-polyethylene LCS patellar component, which may also explain our findings since metal backing was used in all PR LCS TKAs performed in Norway during the study period.

The search for improvement in outcome of TKAs has resulted in the introduction of new designs of both the tibial and the femoral component. Most brands included in this study had a modular fixed-bearing tibial insert, except for the AGC implants where the fixed-bearing tibial component was a metal-backed mono-block and for the LCS, LCS Complete, and e.motion implants where a mobile-bearing system was used. For the LCS implant, both a mobile meniscal bearing and a mobile rotating platform were used. We could not, however, identify any clear advantages related to any of these LCS designs in this study with more than 10 years of follow-up. Further follow-up is needed to determine whether the mobile-bearing knee prostheses lead to lower wear and less loosening. In a recent meta-analysis, no advantage using mobile-bearing knee prostheses was found (Oh et al. 2009), which is supported by our findings.

The shape of the femoral component has been focused on in the search for maximal range of motion and minimal anterior knee pain after TKA. The idea is that the patella needs an anatomic flange and groove in the femoral component to perform satisfactory through bending and stretching; this has been reported to show a clear advantage over the unresurfaced patella regarding complications as well as pain and function (Whiteside and Nakamura 2003). More anatomic femoral components with a narrower fork and deeper groove have been introduced in newer designs, which may explain the better performance of the NR AGC Anatomic, NR Profix, and NR e.motion prostheses as compared to the NR Tricon, NR Genesis I, and NR Duracon. However, the RR estimates for PR Tricon, NR Tricon, and PR Kinemax relative to NR AGC Universal might be overestimated due to failure of the proportional hazards assumption of the Cox model (Schemper 1992). For NR e.motion, the good results may also be caused by the congruent interface between the femoral component and the tibial insert (Morra and Greenwald 2005). This may possibly reduce contact stress and thereby produce less wear. The short follow-up of this prosthesis should be noted, however.

Thus, even though the observed improvement in survival of TKAs over time may to a certain extent be associated with better operation techniques and more experienced surgeons, part of the difference may be explained by the introduction of newer prosthesis brands with better survival that are used in high volume (PR NexGen and NR Profix) together with termination of the use of brands demonstrated to have poorer survival (NR/PR Tricon, NR/PR Genesis I, PR Kinemax, and PR LCS). There were also more recently introduced brands with good short-term results that might have contributed to the increased overall survival of TKAs in the last time period (NR AGC Anatomic and NR e.motion).

### Comparison with other relevant studies

The available evidence regarding patella resurfacing, based on observational studies, RCTs, and meta-analyses, has been summarized in a recent systematic review (Calvisi et al. (2009). While the authors found it difficult to make any strict conclusions due to methodological limitations in the available studies, they found a lower risk of revision and a lower level of postoperative anterior knee pain with the use of resurfaced implants. These advantages were, however, found to be at the expense of potential complications related to the resurfaced patella.

Few studies have assessed the impact of patella resurfacing and of prosthesis brand on implant survival by using data from arthroplasty registers. A previous study from the NAR (Furnes et al. 2002) found a higher risk of revision, but not significantly so, for patella NR implants. The authors did, however, find a significantly higher risk of revision due to pain alone for patella NR TKAs and due to infection for PR TKAs. While the difference in risk of revision due to pain has been confirmed by our findings, we could not demonstrate any difference in risk of revision due to infection. However, by restricting the material to operations performed in a similar time period (from 1994 to 2000) but with longer follow-up (more than 8 years), there was a tendency of a higher risk of revision caused by infection. This tendency was not apparent when considering the operations performed from 2001 to December 10, 2009.

Furnes et al. did not observe significant differences in revision rates among brands, possibly due to short follow-up time. A lower risk of revision of PR implants has also been reported from other arthroplasty registers (the Swedish Knee Arthroplasty Register Annual Report 2009, Clements et al. 2010). In Sweden, PR Kinemax showed poorer survival than PR AGC. NR NexGen performed better than NR AGC, while NR Duracon, NR LCS, and NR Profix performed similarly (the Swedish Knee Arthroplasty Register Annual Report 2009). The NexGen implant has also been found to be the least revised cemented prosthesis brand at 8 years, as reported to AOANJRR (Australian Orthopaedic Association National Joint Replacement Registry Annual Report 2009).

Some of the prosthesis brands included in this study have been examined regarding survival in single-center studies. The PR AGC prosthesis has shown good long-term results (Ritter 2009). These results are in accordance with our findings and they were partly explained by the non-modular tibial component that is thought to offer less backside wear of the polyethylene insert. Positive long-term results have also been demonstrated for uncemented Profix prostheses (Hardeman et al. 2006), but to our knowledge our study is the only one to report 10-year survival results for cemented NR Profix prostheses.

### Strengths and limitations

For economic and practical reasons, comparisons of rare incidences such as revision of joint replacements by use of high-powered RCTs are rarely performed. The main alternative to RCTs is evidence from large observational studies such as arthroplasty registers. Even though it is generally agreed that they are less conclusive, qualitatively speaking results from well-designed observational studies may compare well with those from RCTs (Benson and Hartz 2000). Registry studies may also have advantages such as better external validity due to representation from a wider range of operation procedures, hospitals, implants, patients, and surgeons.

Comparison of survival of different prosthesis designs in observational studies may, however, give results confounded by patient and procedure characteristics. We have treated known confounders (age group, sex, previous operation of the knee, diagnosis, and prosthesis brand—except when comparing brands) by using adjustment in the statistical model. To assess possible effect modification by time of operation, the analyses were also performed stratified by time periods. However, differences in survival may also be confounded by surgeon-related factors and by other variables not reported to the register. The results should therefore be interpreted with caution for brands used in a restricted number of hospitals since skill of the surgeons involved, follow-up routines, and revision threshold may have biased the results. The practice in practically all hospitals in Norway is that implant brand and use or non-use of patella resurfacing are decided for all patients by the medical director of each orthopedics department. Thus, the choice of brand and use of primary patella resurfacing is not normally linked to the surgeon or patient characteristics.

The surgeons can report several reasons for revision simultaneously to the NRL. This could possibly have biased some of our findings regarding specific reasons for revision. An exception is the result of pain alone as a cause of revision.

### Future research

The main argument for resurfacing the patella at the primary TKA is to avoid anterior knee pain and the need of secondary patella resurfacing, at the possible cost of increasing the risk of serious complications related to the patellar component. A recent study from the AOANJRR found more revisions after secondary resurfacing of the patella than after primary insertion of a patellar component (Clements et al. 2010). Since recent studies (Johnston et al. 2009, Lygre et al. 2010) have found no or negligible differences in pain between patients with a primary PR or a patella NR TKA, there should be more investigation of patients' perceptions of the effect of secondary patella resurfacing.

Studies of rare events such as revisions for specific reasons need large numbers of observations. We have studied this for PR and patella NR knee prostheses, and our findings need to be verified by other studies. Studies on differences in reasons for revision related to prosthesis brands are needed to improve the quality of TKA further. The Nordic Arthroplasty Register Association has recently started work with establishment of a common Nordic database in order to pool data from the arthroplasty registers in Denmark, Norway, and Sweden (Robertsson et al. 2010). This could help to minimize the time necessary to gather enough data for investigation of rare events.

A recent study from the NAR has shown a higher risk of revision of TKAs due to infection for patients with rheumatoid arthritis as compared to osteoarthritis of the knee, especially after 5 years of follow-up (Schrama et al. 2010). More knowledge of differences in risk of revision for different diagnoses (and for different prosthesis designs) is still needed, and should be focused on in future studies.

### Possible implications

Our study indicates a need to reconsider the widely accepted recommendation of primary resurfacing of the patella. Less use of a patellar component during primary TKA might be advisable. This will probably give advantages in terms of less extensive operation procedure, shorter duration of the operation (Furnes et al. 2002), better preservation of the soft tissue of the patella, less periprosthetic patella fractures (Chalidis et al. 2007), less total wear of polyethylene, less loosening of the tibial component, and lower cost.

### Conclusion

We found a lower risk of revision of patella resurfaced TKAs compared to patella non resurfaced TKAs, but the difference was not statistically significant. There were, however, differences in reasons for revision. Resurfaced implants had statistically significantly higher risk of revision due to aseptic loosening of the tibial component and due to wear of a polyethylene insert, but had a statistically significantly lower risk of revision due to pain alone. Furthermore, our results might also indicate that the introduction of newer implants and the stop in use of some older inferior designs have improved the survivorship of TKAs in Norway.
